# Mitigating gaseous nitrogen emissions in cotton fields through green manure and reduced nitrogen fertilization

**DOI:** 10.3389/fmicb.2025.1615142

**Published:** 2025-06-02

**Authors:** Ru Ma, Zhenggui Zhang, Jian Wang, Yingchun Han, Ke Li, Mengyao Hou, Yaping Lei, Shiwu Xiong, Beifang Yang, Xiaoyu Zhi, Yahui Jiao, Tao Lin, Shijie Zhang, Yabing Li

**Affiliations:** ^1^Zhengzhou Research Base, State Key Laboratory of Cotton Bio-breeding and Integrated Utilization, School of Agricultural Sciences, Zhengzhou University, Zhengzhou, China; ^2^State Key Laboratory of Cotton Bio-Breeding and Integrated Utilization, Institute of Cotton Research, Chinese Academy of Agricultural Sciences, Anyang, China; ^3^Henan Key Laboratory of Ion-Beam Green Agriculture Bioengineering, Zhengzhou University, Zhengzhou, China; ^4^Xinjiang Academy of Agricultural Sciences, Institute of Cash Crops, Ürümqi, China

**Keywords:** N_2_O emissions, NH_3_ volatilization, green manure, N fertilizer reduction, bacterial keystone taxa

## Abstract

Integrating green manure with reduced nitrogen (N) fertilization is a promising strategy to mitigate N emissions in intensive cotton cultivation, however, the underlying mechanisms remain poorly understood. This study investigated the effects of three green manure incorporation patterns—no green manure (NG), *Orychophragmus violaceus* (OVG), and *Vicia villosa* (VVG)—combined with four N reduction levels (100, 50, 25%, and conventional) on gaseous N emissions (NH_3_ and N_2_O), soil physicochemical properties, and bacterial community characteristics using a cotton field experiment in the Yellow River Basin. Results showed that OVG incorporation with 25% N reduction (N2 treatment) significantly reduced total gaseous N emissions by 36.07% on average during the cotton growth period, reducing NH_3_ and N_2_O emissions by 13.31–54.11% and 32.25–68.77%, respectively, compared with N2 application without OVG. OVG application also increased the relative abundance of Proteobacteria (28.10%), enhanced heterogeneous selection in bacterial community assembly (200%), and increased the complexity of co-occurrence networks, compared with NG. Compared with conventional N fertilization (N3 treatment), ≥50% N reduction significantly lowered NH_3_ (>25.51%) and N_2_O (>32.76%) emissions, reduced the relative abundance of Acidobacteria (−20.23%), simplified co-occurrence networks, and increased homogeneous selection in bacterial assembly (50.00%). Integrating green manure with 25% N reduction substantially reduced gaseous N emissions, which was associated with the enhanced microbial biomass carbon (MBC) and facilitated recruitment of key bacterial taxa (e.g., *Sphingosinicella*, *Azohydromonas*, *Phototrophicus*) within the microbial co-occurrence network. These findings provide insight into how green manure application coupled with N reduction can mitigate gaseous N losses and reshape soil microbial ecology, offering a theoretical basis for sustainable nutrient management during cotton production.

## 1 Introduction

Excessive nitrogen (N) fertilizer application presents major challenges to sustainable agriculture as it leads to nutrient imbalances, soil degradation, and various environmental issues (Qiu et al., 2022; Zhu et al., 2022). Further, inadequate fertilization practices can result in N losses through ammonia (NH_3_) volatilization and nitrous oxide (N_2_O) emissions, thereby exacerbating air quality deterioration and climate change (Rahman Jahangir et al., 2023). NH_3_ volatilization from agricultural systems account for approximately two-thirds of global atmospheric NH_3_ volatilization (IPCC, 2019), with losses of up to 60% of applied N due to volatilization (Wang et al., 2021). N_2_O, a potent greenhouse gas with a global warming potential 298-fold that of CO_2_, is primarily released from agricultural soils and significantly contributes to climate change (IPCC, 2021). Inappropriate management practices, including excessive fertilization and poor application methods, thus exacerbate N losses and increase greenhouse gas emissions (Maaz et al., 2021; Wu et al., 2021). Therefore, improving N use efficiency through optimized fertilization strategies is critical for mitigating environmental pollution.

The potential of organic fertilizers, particularly green manure, to partially substitute chemical N fertilizers has been reported (Li F. et al., 2020; Zhou et al., 2020). Applying green manure can enhance soil N storage and improve crop yield and thus facilitates sustainable agriculture (Ferrara et al., 2021). Leguminous green manures can fix and share atmospheric N with companion crops, thereby enhancing N availability (Adetunji et al., 2020). Non-leguminous green manures help reduce N leaching and improve soil health, which is vital, considering the importance of soil microorganisms (Teixeira et al., 2016; Garland et al., 2021). Furthermore, varying carbon-to-N (C:N) ratios of various green manure types can influence soil microbial community structures and function (Ye et al., 2018; Xu et al., 2024). Incorporating high-C:N-ratio green manures increases bacterial diversity and enzyme activity, whereas low-C:N-ratio green manures enhance N mineralization (Zhou et al., 2020).

Soil microbial communities are critical for nutrient cycling and maintaining soil ecological functions (Yang et al., 2019). In cotton fields, incorporating green manure provides abundant carbon and N sources for soil microorganisms, stimulating microbial proliferation and metabolic activities, thereby affecting the structure and function of the soil microbial community (Liu et al., 2022). The incorporation of green manure also enhances soil porosity and aeration, improves soil structure, and creates favorable conditions for the growth and activity of aerobic microorganisms, ultimately altering the composition and diversity of soil microbial communities (Bungau et al., 2021; Lyu et al., 2023). Concurrently, N fertilizer application exerts significant impacts on soil microbial communities. Within an appropriate threshold range, moderate N application can stimulate microbial growth and reproduction, increasing microbial biomass and diversity. However, excessive N application may lead to structural imbalance in microbial communities (Ren et al., 2020; Ma et al., 2021). The integration of N reduction with green manure incorporation represents a promising management strategy for cotton fields. Although existing studies have confirmed the synergistic effects of this combined approach on soil microorganisms, further in-depth exploration is required to elucidate the underlying mechanisms of community succession and regulatory pathways for key functional microbial groups, particularly core microbial taxa involved in nutrient cycling.

Therefore, we investigated the combined impact of two types of green manure—*Orychophragmus violaceus* (OVG) and *Vicia villosa* (VVG)—combined with four N reduction treatments: 100% reduction (N0), 50% reduction (N1), 25% reduction (N2), and conventional N application (N3) on gaseous N emissions (NH_3_ and N_2_O), soil physicochemical properties, and bacterial communities in cotton fields in the Yellow River Basin. We aimed to elucidate the response characteristics of gaseous N emissions to green manure application coupled with reduced N fertilization, in addition to the responses of soil bacterial communities.

## 2 Materials and methods

### 2.1 Site description and experimental design

The experimental site was located at the Anyang Experimental Station (36°06′N, 114°21′E; 76 m above sea level) in northern China. This region is at the center of the cotton growing area in the Yellow River Basin, where monoculture cotton is the predominant cropping system.

The experiment was conducted during the 2022 and 2023 cotton growing seasons, and cotton was planted on April 24 and harvested on November 2. A split-plot design was employed, with green manure incorporation as the main plot factor, i.e., no green manure (NG), incorporation of *Orychophragmus violaceus* (OVG), and incorporation of *Vicia villosa* (VVG). The subplot factor was four N levels, i.e., N3 (225 kg ha^−1^, local N application rate in the Yellow River Basin cotton area), N2 (168.75 kg ha^−1^, a 25% reduction compared with N3), N1 (112.50 kg ha^−1^, a 50% reduction compared to N3), and N0 (0 kg ha^−1^, a 100% reduction compared with N3). The experiment comprised 12 treatments with three replications each, totaling 36 plots, each with an area of 64 m^2^ (8 m × 8 m). The cotton cultivar “CCRI 60” was used at a planting density of 9 × 10^5^ plants ha^−1^. The green manure crops OVG (C:N ratio = 41.8:1) and VVG (C:N ratio = 15.3:1) were sown during the cotton boll-opening stage (mid-September) each year. At full bloom stage in the following mid-April, the biomass of green manure crops was measured, balanced to an incorporation rate of 4,500 kg ha^−1^, chopped into >5 cm pieces, and incorporated into the soil using a rotary tiller. Fertilizers used in the experiment were urea (46.4% N), superphosphate (46% P_2_O_5_), and potassium sulfate (52% K_2_O). N fertilizer was applied in two splits: 50% as basal and 50% as top-dressing at the initial flowering stage. Phosphate and potassium fertilizers were applied as basal applications at a rate of 102 kg ha^−1^ each. Other agronomic practices followed local high-yield cotton field management practices.

### 2.2 Ammonia (NH_3_) collection and measurement

NH_3_ volatilization was measured using the ventilation chamber method combined with the indophenol blue colorimetric method (Yang et al., 2015). Specifically, a PVC sampling cylinder (10 cm high, 15 cm diameter) with open ends was inserted into the soil, containing two sponges soaked in phosphoglycerol solution. The upper sponge, aligned with the top of the PVC cylinder, served as an air isolation layer, while the lower sponge, positioned 4 cm above the soil surface, absorbed volatilized NH_3_. During sampling, the lower sponge was removed and stored in a sealed bag, while a new phosphoglycerol-soaked sponge was placed inside. The upper sponge was replaced every 3–7 d. The sponge was extracted with 1 mol L^−1^ KCl solution, and the extract was analyzed for NH_3_ within 24 h. NH_3_ volatilization was sampled at 16:00 on days 1, 2, 3, 4, 5, 6, 7, 10, 13, 20, 27, and 34 after fertilization, with 24 samplings during the cotton growing season in 2022 and 2023, respectively. NH_3_ flux and cumulative volatilization were calculated according to the following:


$$F_{\mathrm{NH}3}=\frac{C\times V}{A\times D}\times 10^{-2}$$

where F_NH3_ is the NH_3_ flux (kg NH_4_^+^-N hm^−2^ d^−1^), C is the concentration of NH_4_^+^-N in the extract (mg L^−1^), V is the volume of the extract (L), A is the cross-sectional area of the ventilation chamber (m^2^), and D is the interval between sampling dates (d).


$$CF_{\mathrm{NH}3}=\frac{1}{2}\times \sum_{i=1}^{n}[(F_{i}+F_{i-1})\times (T_{i}-T_{i-1})]$$

where CF_NH3_ is the cumulative NH_3_ volatilization (kg NH_4_^+^-N hm^−2^), *n* is the total number of samplings, F_i_ is the NH_3_ flux of *i*th sampling, and T_i_ is the time (d) of *i*th sampling.

### 2.3 Nitrous oxide (N_2_O) collection and measurement

N_2_O emissions were monitored using static chamber-gas chromatography (Liu et al., 2019). The static chamber system consisted of an organic glass base and a sampling chamber (20 cm × 20 cm × 29.5 cm) with a water trough along the base’s outer edge. During sampling, the chamber was placed on the base, and water was added to the trough to prevent atmospheric gas exchange. Gas samples were collected using a 40 mL plastic syringe at 15, 30, 45, and 60 min after sealing the chamber and analyzed using a gas chromatograph (7890B, Agilent Technologies, USA) to determine N_2_O concentrations. Sampling was conducted weekly during the seedling, squaring, and flowering stages, and biweekly during the boll opening stage, between 9:00 and 11:00 a.m., with 22 samplings in 2022 and 2023, respectively. N_2_O flux and cumulative emissions were calculated according to the following:


$$F_{N2O}=\rho \times \frac{P}{P_{0}}\times \frac{V}{A}\times \frac{273}{273+T}\times \frac{\mathrm{\Delta c}}{\mathrm{\Delta t}}$$

where F_N2O_ is the N_2_O flux (μg m^−2^ h^−1^), *ρ* is the density of the gas under standard conditions (kg m^−3^), P is the pressure inside the sampling chamber, P_0_ is the standard atmospheric pressure, V is the volume of the chamber (m^3^), A is the cross-sectional area of the chamber (m^2^), T is the average temperature inside the chamber during measurement (°C), and Δc/Δt is the rate of change in N_2_O concentration over time.


$$CF_{N2O}=\frac{1}{2}\times 24\times 10^{-5}\times \sum_{i=1}^{n}[(F_{i}+F_{i-1})\times (T_{i}-T_{i-1})]$$

where CF_N2O_ is the cumulative N_2_O emissions (kg N ha^−1^), *n* is the total number of samplings, F_i_ is the N_2_O (μg m^−2^ h^−1^) flux of *i*th sampling, and T_i_ is the time (d) of *i*th sampling.

### 2.4 Soil sampling and physiochemical analysis

Soil samples were collected from the 0–20 cm soil layer at the cotton harvest stage in 2023. Five samples were taken from each plot using an “S” shaped pattern and were mixed thoroughly before passing through a 2-mm sieve to remove plant roots and stones. The sieved samples were divided into three portions: one portion was stored at 4°C for nitrate N (NO_3_^−^-N), ammonium N (NH_4_^+^-N), microbial biomass C (MBC), and microbial biomass N (MBN) measurements; the second portion was air-dried and sieved through 20- and 100-mesh screens for available phosphorus (AP), available potassium (AK), pH, soil organic C (SOC), and total N (TN) analysis; the third portion was stored at −80°C for subsequent molecular biological analysis.

NO_3_^−^-N and NH_4_^+^-N were determined using dual-wavelength UV spectrophotometry, with NH_4_^+^-N measured by indophenol blue colorimetry (UV2450, Shimadzu, Japan). MBC and MBN were assessed using the chloroform fumigation extraction method, with C determined using a total organic C analyzer (TOC-VCPH, Shimadzu, Japan) and N using a continuous flow analyzer (Skalar San++, Skalar, Netherlands). AP was measured using the molybdenum-antimony anti-colorimetry method. AK was determined using atomic absorption spectrophotometry (ZEEnit 700 P, Jena, Germany). TN and SOC were determined using the combustion method, with samples analyzed using an elemental analyzer (Elementar Vario MACRO, Elementar Germany). Soil pH was measured using a pH meter (LE438PH, Mettler Toledo, Germany) in a 1:2.5 soil-to-water suspension.

### 2.5 High-throughput sequencing and analysis

Total genomic DNA was extracted from soil samples using the TGuide S96 Magnetic Soil/Feces DNA Kit (Tiangen Biotech, Beijing, China). The quality and quantity of the extracted DNA were measured using a NanoDrop 2000 UV–Vis spectrophotometer (Thermo Scientific, Wilmington, USA). The V3-V4 hypervariable regions of the bacterial 16S rRNA gene were amplified using the primer pair 338F (5’-ACTCCTACGGGAGGCAGCA-3′) and 806R (5’-GGACTACHVGGGTWTCTAAT-3′). PCR amplification was performed in a 10 μL total reaction volume containing 5–50 ng of template DNA, 0.3 μL of each primer (10 μM), 5 μL of KOD FX Neo buffer, 2 μL of dNTPs (2 mM each), 0.2 μL of KOD FX Neo polymerase, and ddH_2_O up to 20 μL. The thermal cycling conditions were as follows: an initial denaturation at 95°C for 5 min, followed by 30 cycles of denaturation at 95°C for 30 s, annealing at 50°C for 30 s, extension at 72°C for 40 s, and a final extension at 72°C for 7 min. The PCR products were purified using an Omega DNA Purification Kit (Omega Inc., Norcross, GA, USA) and quantified using a Qsep-400 system (BiOptic, New Taipei City, Taiwan, ROC). The amplicon libraries were sequenced on an Illumina NovaSeq 6,000 platform with 2 × 250 bp paired-end reads (Biomarker Technologies Co., Ltd., Beijing, China).

Raw paired-end reads were processed using Qiime2. Most forward and reverse reads were merged to form a complete sequence. Low-quality reads and noise were removed using the DADA2 plugin to obtain amplicon sequence variants (ASVs) (Callahan et al., 2016). The ASVs were then classified and annotated via comparison with the SILVA database (Release 138) using the mothur software (Schloss et al., 2009). All sequences have been uploaded to the NCBl Sequence Read Archive under accession number PRNA1111008.

### 2.6 Statistical analyses

All data were tested for homogeneity of variance using Levene’s tests before further analysis. One-way analysis of variance and the least-significant difference test were used to compare differences in NH_3_ and N_2_O emissions, cotton yield and yield components, soil physicochemical properties, and microbial alpha diversity indices among treatments (IBM SPSS Statistics 27). Non-metric multidimensional scaling (NMDS) based on Bray-Curtis distances was performed using the “vegan” package in R software to analyze soil bacterial community structure differences among treatments. The R package “ggtern” and STAMP 2.1.3 were employed to analyze differences in bacterial community composition among treatments.

A neutral community model (NCM) was employed to evaluate the role of stochastic processes in the assembly of bacterial communities under different treatments. To further elucidate the ecological processes governing bacterial community assembly, the beta-nearest taxon index (*β*-NTI) and Raup-Crick index based on Bray–Curtis dissimilarity (RC_bray_) were calculated using a null model with the R package “picante” (Stegen et al., 2013). In β-NTI metrics, |β-NTI| < 2 indicated a stochastic process; |β-NTI| > 2 indicated a deterministic process; |β-NTI| < 2 and RC_bray_ > 0.95 indicated diffusion limitation; |β-NTI| < 2 and RC_bray_ < −0.95 indicated uniform diffusion; and |β-NTI| < 2 and |RC_bray_| < 0.95 indicated undominated processes such as ecological drift may dominate (Stegen et al., 2012). In addition, ecotype width indices were calculated using functions from the R package “spaa” to explore the relative effects of stochastic and deterministic processes of bacterial communities.

Molecular ecological network analysis was conducted to construct co-occurrence networks, which were visualized using Gephi 0.9.2 (Chen J. et al., 2020). The keystone amplicon sequence variants within the network were identified in terms of within-module connectivity (Zi) and among-module connectivity (Pi) as follows: network hubs, Zi > 2.5 and Pi > 0.62; module hubs, Zi > 2.5 and Pi < 0.62; and connectors, Zi < 2.5 and Pi > 0.62 (Shi et al., 2016). To elucidate the relationships between soil parameters and NH_3_ and N_2_O emissions, correlation analysis, random forest analysis, and partial least-squares path modeling (PLS-PM) were performed using the R packages “corrplot,” “rfPermute,” and “plspm,” respectively. An overall model goodness-of-fit of PLS-PM > 0.60 was considered acceptable (Chen et al., 2022).

## 3 Results

### 3.1 Gaseous N emissions

**Figure 1:**
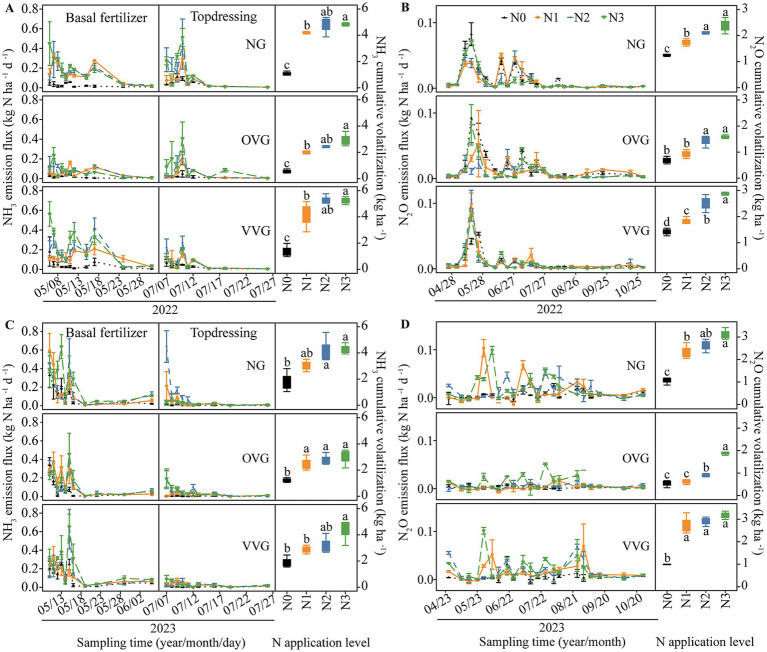
Effects of green manure incorporation and N fertilizer reduction on NH_3_ **(A,C)** and N_2_O **(B,D)** emissions in cotton fields. Error bars represent the standard error of the mean. Different letters indicate significant differences (*p* < 0.05) among N treatments. NG, no incorporation (control); OVG, incorporation of *Orychophragmus violaceus*; VVG, incorporation of *Vicia villosa*; N3, maximum economic N rate; N2, 25% N reduction compared to N3; N1, 50% N reduction compared to N3; N0, no N application.

The incorporation of green manure coupled with N fertilizer reduction significantly influenced soil gaseous N emissions (Figure 1). NH_3_ volatilization peaked immediately after each fertilization event and decreased over time (Figures 1A,C). In 2022 and 2023, cumulative NH_3_ emissions ranged from 1.08 to 5.25 kg ha^−1^ for NG, from 0.56 to 2.98 kg ha^−1^ for OVG, and from 1.38 to 5.65 kg ha^−1^ for VVG. Compared with NG, OVG significantly reduced NH_3_ emissions by 28.94–48.16% (*p* < 0.05), and VVG showed an increase of 7.52% in 2022 (*p* > 0.05) and a decrease of 7.47% in 2023 (*p* < 0.05). From 2022 to 2023, cumulative NH_3_ emissions decreased significantly with reduced N application. Compared with N3, N2 reduced NH_3_ emissions by 9.95–11.81% (*p* > 0.05), whereas N1 and N0 reduced emissions by 25.51–27.78% and 57.34–78.17%, respectively (*p* < 0.05). A significant interaction effect between green manure incorporation and N application on NH_3_ volatilization was observed in 2022 (Supplementary Table 1).

N_2_O emissions varied significantly across treatments, peaking during the cotton seedling stage in May before gradually declining to baseline levels (Figures 1B,D). In 2022 and 2023, cumulative N_2_O emissions ranged from 1.06 to 3.12 kg ha^−1^ for NG, from 0.54 to 1.88 kg ha^−1^ for OVG, and from 0.99 to 3.49 kg ha^−1^ for VVG. Compared with NG, OVG significantly reduced cumulative N_2_O emissions by 38.10–56.96% from 2022 to 2023, while VVG increased emissions by 10.88–16.09% (*p* > 0.05). Compared with N3, N2 reduced N_2_O emissions by 12.27–24.28% (*p* > 0.05), whereas N1 and N0 reduced emissions by 32.76–34.88% and 51.43–69.55%, respectively (*p* < 0.05). A significant interaction effect between green manure incorporation and N application on N_2_O emissions was observed in 2023 (Supplementary Table 1).

Overall, cumulative gaseous N emissions in 2022 and 2023 ranged from 2.33 to 7.62 kg ha^−1^ for NG, 1.24–4.87 kg ha^−1^ for OVG, and 2.80–8.58 kg ha^−1^ for VVG (Supplementary Table 1). OVG significantly reduced total gaseous N emissions by 40.33–44.86% compared with NG (*p* < 0.05). N1 and N0 reduced emissions by 28.62–29.88% and 62.50–69.29% (*p* < 0.05) compared with N3. Notably, OVG incorporation combining N2 fertilization significantly reduced total gaseous N emissions by 36.07% on average compared to N2 without OVG, among which NH_3_ and N_2_O emissions were reduced by 13.31–54.11% and 32.25–68.77%, respectively. The interaction between green manure incorporation and N application on gaseous N emissions was significant in both years (Supplementary Table 1).

### 3.2 Soil physicochemical properties

Green manure incorporation and N fertilizer reduction significantly influenced soil physicochemical properties; however, their interaction had limited effects (Table 1). Specifically, compared with NG, OVG incorporation significantly increased soil pH, MBN, and MBC by 1.36, 68.47, and 79.27%, respectively, but reduced NH_4_^+^-N (30.71%) and NO_3_^−^-N (65.21%; *p* < 0.05). In contrast, VVG incorporation significantly increased pH (1.06%), and MBC (80.13%), compared with NG, and no significant change was observed in other soil properties. Compared with N3, N reduction (N2, N1, N0) produced significantly reduced NH_4_^+^-N, NO_3_^−^-N, MBN, and AP contents with averages of 22.83, 35.24, 32.49, and 30.18%, respectively. Higher N application decreased soil MBC, and a statistically significant interaction effect was observed between N level and green manure treatments.

**Table 1:** Soil physiochemical properties in response to green manure incorporation and nitrogen reduction in cotton fields.

Treatment	pH	NH_4_^+^-N (mg kg^−1^)	NO_3_^−^-N (mg kg^−1^)	MBN (mg kg^−1^)	MBC (mg kg^−1^)	SOC (g kg^−1^)	TN (g kg^−1^)	AP (mg kg^−1^)	AK (mg kg^−1^)
NGN0	8.36 ± 0.04Ba	8.26 ± 0.75Bb	7.41 ± 2.06Ac	1.67 ± 0.46Bb	82.5 ± 2.45Ba	10.7 ± 0.21Ba	0.51 ± 0.13Aa	9.87 ± 1.06Aa	136 ± 18.0ABa
NGN1	8.27 ± 0.03Bb	9.44 ± 1.22Bb	8.22 ± 0.72Abc	2.24 ± 0.19Bab	70.0 ± 10.1Bab	13.5 ± 2.78Ba	0.52 ± 0.02Aa	10.1 ± 5.96Aa	119 ± 16.4ABa
NGN2	8.16 ± 0.03Bc	11.3 ± 0.75Ba	11.1 ± 1.72Aab	2.90 ± 0.90Bab	62.0 ± 4.93Bb	12.4 ± 0.89Ba	0.60 ± 0.23Aa	12.7 ± 2.18Aa	125 ± 17.0ABa
NGN3	8.15 ± 0.04Bc	12.4 ± 0.94Ba	13.9 ± 2.44Aa	3.53 ± 1.20Ba	48.3 ± 8.03Bc	11.8 ± 2.42Ba	0.65 ± 0.15Aa	13.1 ± 4.33Aa	126 ± 12.9ABa
OVGN0	8.38 ± 0.02Aa	5.47 ± 1.19Bc	1.29 ± 0.58Bc	2.94 ± 0.85Ab	131 ± 16.2Aa	10.9 ± 1.90Aa	0.56 ± 0.09Aa	11.6 ± 1.29Ab	125 ± 18.8Ab
OVGN1	8.37 ± 0.04Aa	6.75 ± 1.02Bbc	2.96 ± 0.61Bb	4.27 ± 0.58Aab	122 ± 8.15Aab	14.0 ± 1.83Aa	0.59 ± 0.09Aa	10.4 ± 2.16Ab	123 ± 10.0Ab
OVGN2	8.34 ± 0.04Aab	7.46 ± 0.54Bab	4.56 ± 0.92Ba	4.72 ± 0.92Aa	108 ± 9.69Ab	14.0 ± 1.26Aa	0.64 ± 0.14Aa	11.9 ± 2.56Ab	168 ± 35.3Aa
OVGN3	8.29 ± 0.04Ab	8.99 ± 0.46Ba	5.31 ± 0.57Ba	5.51 ± 0.40Aa	110 ± 5.02Ab	12.8 ± 2.01Aa	0.63 ± 0.03Aa	16.9 ± 3.68Aa	137 ± 12.5Aab
VVGN0	8.41 ± 0.03Aa	8.29 ± 0.36Ac	7.21 ± 1.02Ab	1.85 ± 0.70Ba	127 ± 13.4Aa	11.5 ± 0.70Aa	0.54 ± 0.04Aa	7.87 ± 1.55Aa	114 ± 7.70Ba
VVGN1	8.30 ± 0.02Ab	10.4 ± 0.59Ab	7.96 ± 1.24Ab	2.40 ± 0.67Ba	118 ± 12.0Aa	13.1 ± 1.44Aa	0.62 ± 0.16Aa	12.1 ± 5.78Aa	115 ± 10.2Ba
VVGN2	8.28 ± 0.08Ab	12.2 ± 0.59Aa	11.2 ± 1.71Aa	2.76 ± 0.88Ba	118 ± 6.78Aa	14.1 ± 1.83Aa	0.66 ± 0.11Aa	10.8 ± 0.40Aa	128 ± 18.4Ba
VVGN3	8.29 ± 0.03Ab	13.0 ± 1.56Aa	12.6 ± 1.62Aa	3.66 ± 1.40Ba	111 ± 16.8Aa	11.6 ± 1.69Aa	0.62 ± 0.10Aa	16.5 ± 9.00Aa	123 ± 16.5Ba
GM	***	***	***	***	***	*	ns	ns	ns
NF	***	***	***	***	***	ns	ns	*	ns
GM*NF	***	ns	ns	ns	*	ns	ns	ns	ns

Data are presented as mean ± standard error (*n* = 3). Uppercase letters indicate significant differences at *P* < 0.05 according to the least-significant difference test among different green manure incorporation treatments (GM), and lowercase letters indicate significant differences among nitrogen fertilization levels (NF). NG, no incorporation (control); OVG, incorporation of *Orychophragmus violaceus*; VVG, incorporation of *Vicia villosa*; N3, maximum economic nitrogen rate; N2, 25% nitrogen reduction compared to N3; N1, 50% nitrogen reduction compared to N3; N0, no nitrogen application. NH_4_^+^-N, ammonia nitrogen; NO_3_^−^-N, nitrate nitrogen; MBN, microbial biomass nitrogen; MBC, microbial biomass carbon; SOC, soil organic carbon; TN, total nitrogen; AP, available phosphorus; AK, available potassium. Asterisks denote statistical significance: *p < 0.05, **p < 0.01, ***p < 0.001; “ns” indicates not significant.

### 3.3 Bacterial community composition and diversity

**Figure 2:**
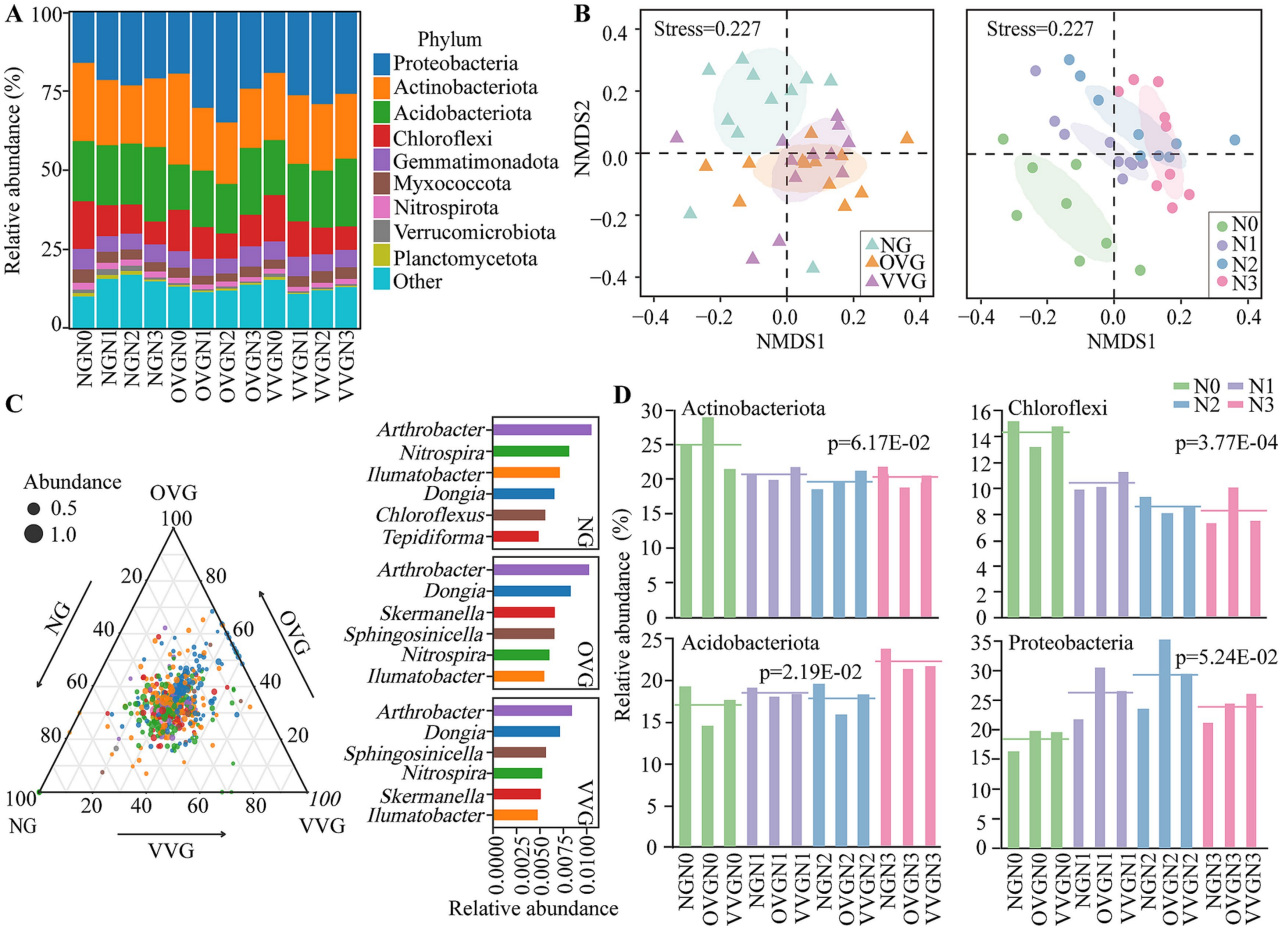
Effects of green manure incorporation and N fertilizer reduction on bacterial community composition and diversity. **(A)** Relative abundance of soil bacterial communities at the phylum level. **(B)** Nonmetric multidimensional scaling (NMDS) analysis showing the beta diversity of the soil bacterial communities. The ellipses represent 50% confidence intervals. **(C)** Ternary plot illustrating the distribution of the top 500 amplicon sequence variants (ASVs) responding to green manure patterns, with the most enriched genera displayed (*p* < 0.05, *n* = 12). **(D)** Bar charts showing significant phyla responding to varying N fertilization levels (*p* < 0.05, *n* = 9). NG, no incorporation (control); OVG, incorporation of *Orychophragmus violaceus*; VVG, incorporation of *Vicia villosa*; N3, maximum economic N rate; N2, 25% N reduction compared to N3; N1, 50% N reduction compared to N3; N0, no N application.

The composition and diversity of soil bacterial communities were significantly affected by green manure incorporation and N fertilizer reduction. The dominant bacterial phyla across all treatments were Proteobacteria (24.31%), Actinobacteria (21.35%), Acidobacteriota (18.70%), and Chloroflexi (10.34%; Figure 2A). NMDS analysis of beta diversity revealed significant shifts in bacterial community structures across treatments, with a clear separation between NG and the green manure treatments (OVG and VVG) as well as among the different N levels (Figure 2B).

Compared with NG, OVG significantly increased the relative abundance of Proteobacteria by 28.1%, whereas VVG significantly decreased that of Acidobacteriota by 16.57% (*p* < 0.05; Figure 2C). The dominant genera also varied with green manure treatments, e.g., *Sphingosinicella* and *Skermanella* were enriched under VVG and OVG treatments, respectively (Figure 2C). With decreasing N levels (from N3 to N0), the relative abundance of Acidobacteriota significantly decreased by 20.23%, whereas that of Chloroflexi increased by 34.53% (*p* < 0.05; Figure 2D). At the genus level, *Thermomarinilinea* (Chloroflexi), *Thiobacter* (Proteobacteria), and *Nocardioides* (Actinobacteriota) increased by 33.49, 10.77, and 7.71%, respectively, with reduced N application. In contrast, *Usitatibacter* (Proteobacteria) showed a significant decrease of 3.98% (*p* < 0.05; Supplementary Figure 1). Alpha diversity indices, including ACE, Chao1, and Shannon, generally increased with green manure incorporation; however, these indices did not differ significantly across N addition levels (Supplementary Figure 2).

### 3.4 Bacterial community assembly and co-occurrence networks

**Figure 3:**
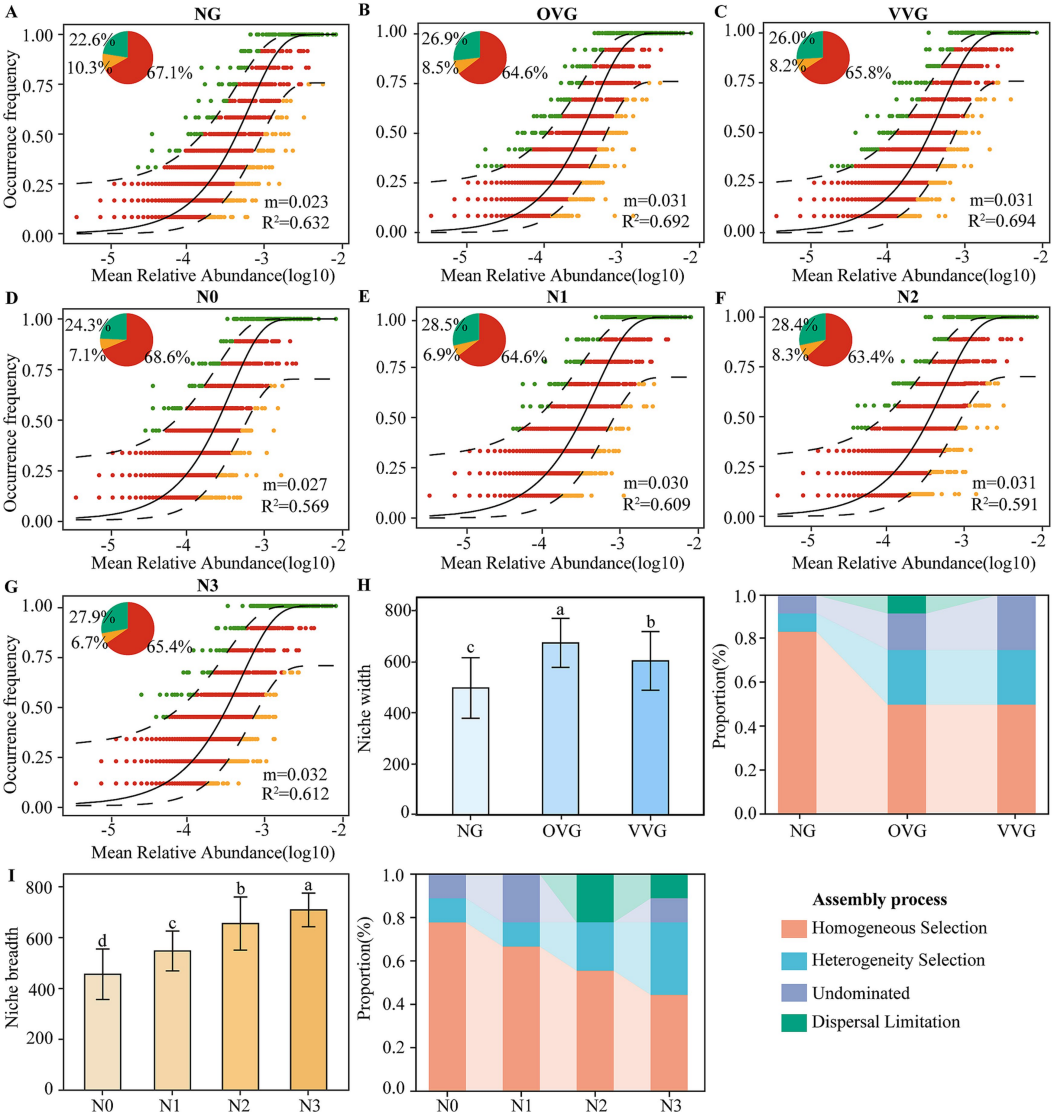
Bacterial community assembly processes and niche breadths in response to green manure incorporation and N fertilizer reduction. Fit of the neutral community models of bacterial taxa under patterns of **(A)** NG, **(B)** OVG, **(C)** VVG, **(D)** N0, **(E)** N1, **(F)** N2, and **(G)** N3. Niche breadth differences and relative contributions of deterministic and stochastic processes under patterns of green manure incorporation **(H)** and nitrogen fertilizer reduction **(I)**. ASVs represented by green dots showed a higher frequency of occurrence than the model predicts, and those represented by orange dots were the opposite. ASVs predicted by the 95% confidence interval of the model are shown as red dots. m is the migration rate between populations, and *R*^2^ indicates the fit to this model. NG, no incorporation (control); OVG, incorporation of *Orychophragmus violaceus*; VVG, incorporation of *Vicia villosa*; N3, maximum economic N rate; N2, 25% N reduction compared to N3; N1, 50% N reduction compared to N3; N0, no N application.

NCM analysis indicated a moderate influence of stochastic processes in bacterial community assembly across treatments, and R^2^ values ranged from 0.57 to 0.69 (Figures 3A–G). Incorporation of green manure (OVG and VVG) and higher N levels (N2 and N3) reduced stochastic influences, as evidenced by higher migration rates (m values of 0.031–0.032). Phylogenetic null model results further confirmed the dominant role of deterministic processes, particularly with regard to homogeneous selection (44.44–83.33%). In contrast to NG, green manure treatments produced a notable increase in heterogeneous selection (averaging 200%). Reduced N fertilization led to a significant increase in homogeneous selection (from 44.44 to 77.78%) and a decrease in heterogeneous selection (from 33.30 to 11.11%) and in dispersal limitation (from 11.11 to 0%). Niche breadth analysis revealed that ecological niches were broader in OVG (706.38) and VVG (601.46) than in NG (495.73; Figure 3H, *P* < 0.05). Conversely, reducing N levels (N3 to N0) significantly narrowed niche widths from 746.57 to 455.71 (Figure 3I).

**Figure 4:**
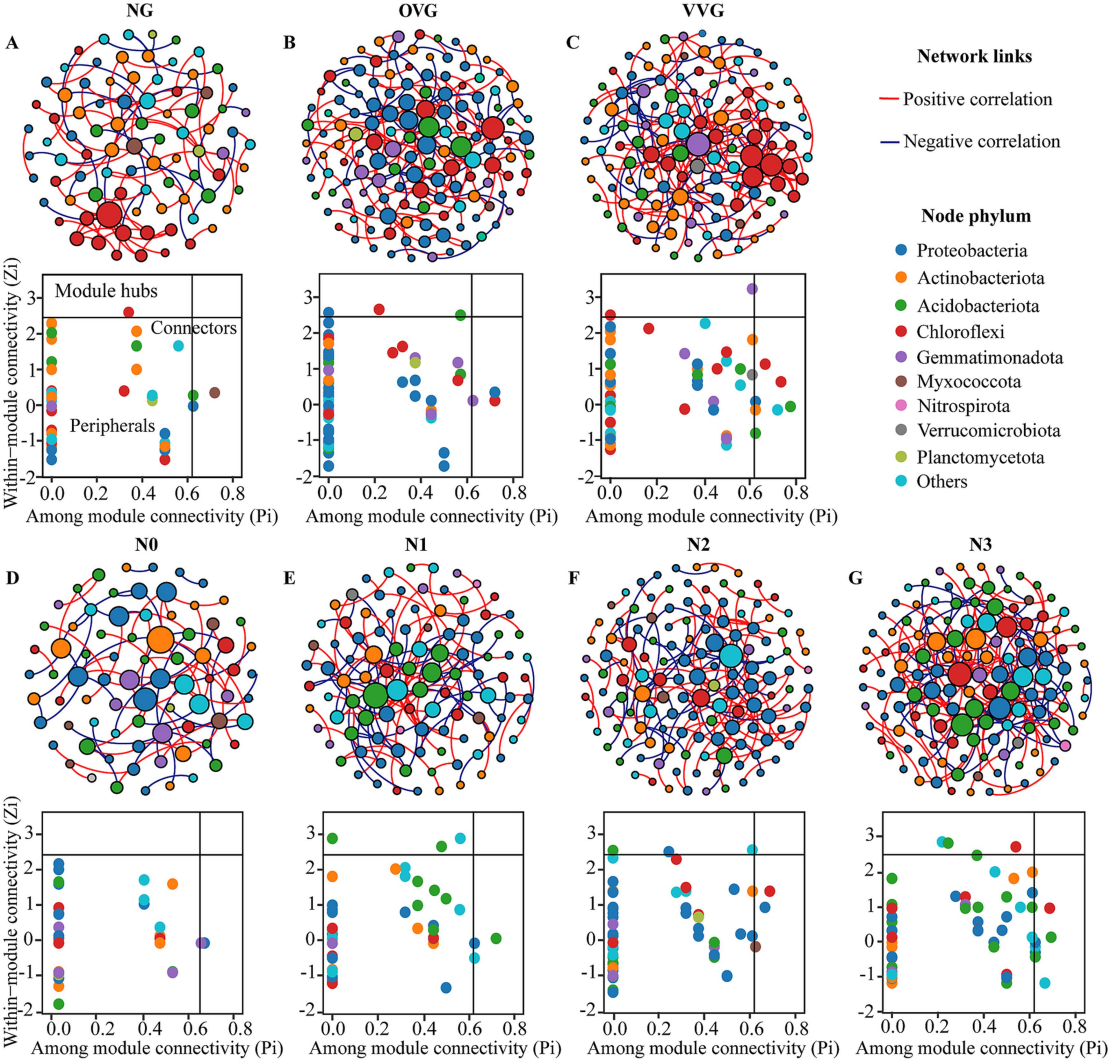
Co-occurrence networks analysis of bacterial communities in response to green manure incorporation and N fertilizer reduction. Co-occurrence networks and distributions of keystone taxa under patterns of **(A)** NG, **(B)** OVG, **(C)** VVG, **(D)** N0, **(E)** N1, **(F)** N2, and **(G)** N3. The size of each node in networks is proportional to the number of degrees. Keystone taxa in each network identified by within-module connectivity (Zi) and among-module connectivity (Pi). The nodes were colored at the phylum level. NG, no incorporation (control); OVG, incorporation of *Orychophragmus violaceus*; VVG, incorporation of *Vicia villosa*; N3, maximum economic N rate; N2, 25% N reduction compared to N3; N1, 50% N reduction compared to N3; N0, no N application.

Co-occurrence network analysis demonstrated increased complexity in the OVG and VVG treatments, characterized by a greater number of nodes, edges, and higher clustering coefficients, indicating a more interconnected community structure (Figure 4, Table 2). In contrast, N reduction led to simpler networks with fewer nodes and edges, reflecting reduced microbial interactions. Zi-Pi analysis identified distinct keystone taxa across treatments. The OVG and VVG treatments supported a wider range of keystone taxa, primarily including the genera *Sphingomonas*, *Azohydromonas*, and *Phototrophicus*. The number of keystone taxa decreased with decreasing N levels from N3 to N0. Keystone species at the highest N level (N3) were predominantly from genera such as *Luteitalea* and *Holophaga* of the phylum Acidobacteriota (Supplementary Table 2).

**Table 2:** Topological properties of different bacterial community co-occurrence networks.

Network Index	Green manure pattern			N fertilization level			
	NG	OVG	VVG	N0	N1	N2	N3
Number of nodes	86	138	124	77	102	134	132
Number of edges	101	170	184	73	122	165	187
(Positive/Negative)	(70.3%/29.7%)	(69.4%/30.6%)	(69.0%/31.0%)	(48.0%/52.1%)	(60.7%/39.3%)	(68.5%/31.5%)	(63.6%/36.4%)
Average clustering coefficient	0.06	0.08	0.07	0.00	0.04	0.15	0.06
Average path distance	5.85	7.12	4.44	4.98	4.94	4.52	4.18
Modularity	0.71	0.75	0.64	0.73	0.70	0.71	0.62
Geodesic efficiency	0.22	0.21	0.27	0.27	0.25	0.27	0.28
Connectedness	0.91	0.67	0.94	0.45	0.83	0.56	0.79

NG, no incorporation (control); OVG, incorporation of *Orychophragmus violaceus*; VVG, incorporation of *Vicia villosa*; N3, maximum economic nitrogen rate; N2, 25% nitrogen reduction compared to N3; N1, 50% nitrogen reduction compared to N3; N0, no nitrogen application.

### 3.5 Associations of gases N emissions, bacterial community, and soil properties

**Figure 5:**
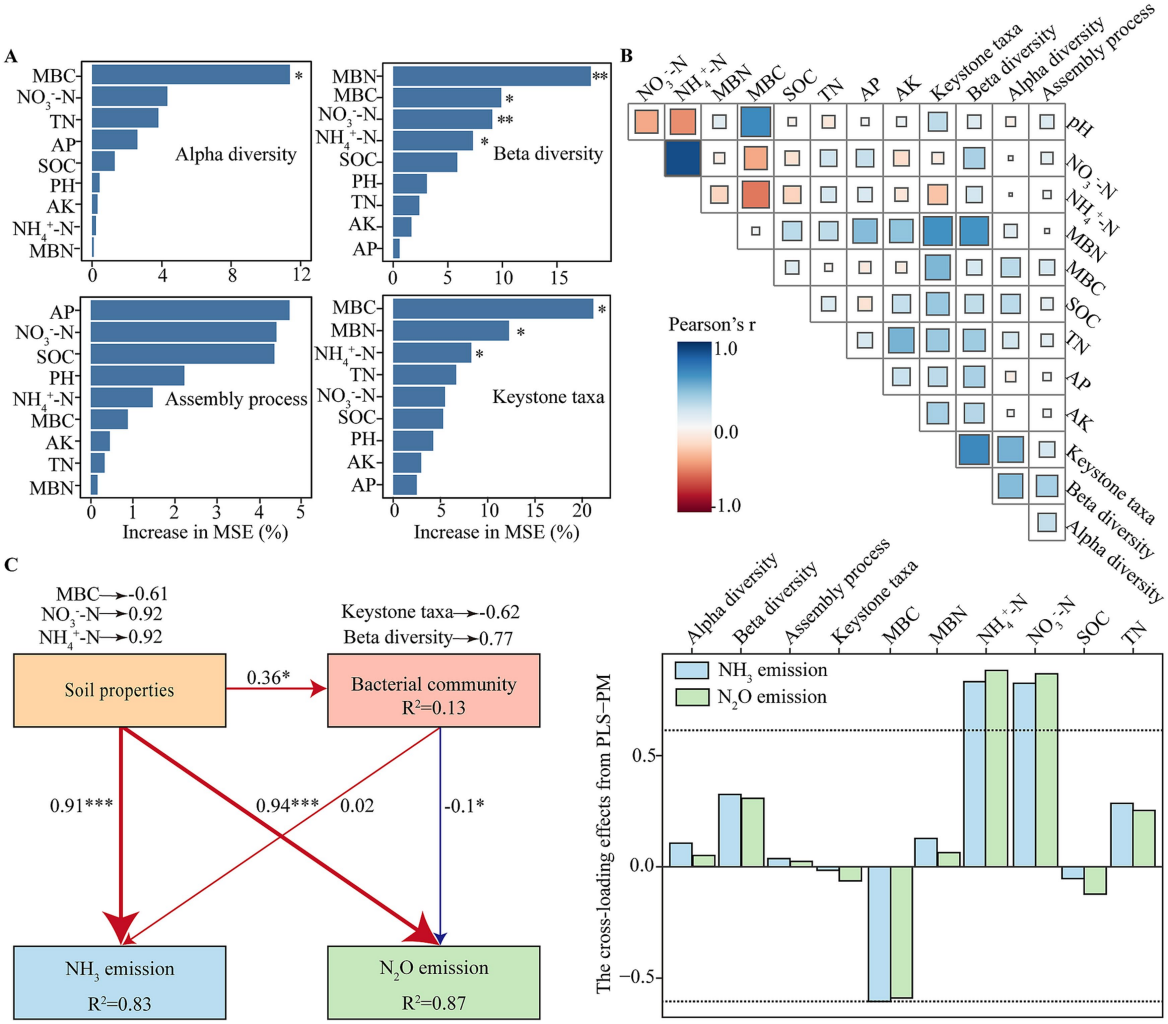
Relationships between soil physicochemical properties and bacterial communities, and their potential contributions to gases N emissions. **(A)** Random forest model (RFM) revealing importance of soil properties influencing bacterial community diversity, assembly, and keystone taxa. **(B)** Pearson correlation matrix showing relationships among soil biotic and abiotic parameters. **(C)** Partial least squares-path modeling (PLS-PM) illustrating the direct and indirect effects of significant soil properties and bacterial communities on NH_3_ and N_2_O emissions. Path coefficients are indicated next to the arrows; line thickness is proportional to coefficient values. Red and blue arrows indicate positive and negative effects, respectively. Asterisks denote statistical significance at * *p* < 0.05, **p < 0.01, and ***p < 0.001. The cross-loading values for all parameters displayed by the bar charts; factor loadings above 0.6 are represented by dashed lines.

The interactions among soil properties, bacterial communities, and N emissions were evaluated using random forest modeling (RFM), Pearson correlation analysis, and PLS-PM (Figure 5). RFM and Pearson’s correlation analysis identified soil properties, including MBN, MBC, NO_3_^−^-N, and NH_4_^+^-N, as critical factors influencing bacterial community diversity, assembly, and keystone taxa (Figures 5A,B).

The PLS-PM model revealed that soil properties had a significant direct positive effect on both NH_3_ (path coefficient = 0.91, *p* < 0.05) and N_2_O emissions (path coefficient = 0.94, *p* < 0.05). Among these soil properties, NO_3_^−^-N (loading = 0.92), NH_4_^+^-N (loading = 0.92), and MBC (loading = −0.61) were the predominant contributors affecting N emissions. Additionally, soil properties indirectly influenced NH_3_ and N_2_O emissions by modulating bacterial communities (path coefficient = 0.36). Bacterial communities exhibited a non-significant positive direct effect on NH_3_ emissions (path coefficient = 0.02) and a significant negative direct effect on N_2_O emissions (path coefficient = −0.10). Within the microbial attributes, beta diversity (loading = 0.77) and keystone taxa (loading = −0.62) were identified as primary contributors, and keystone taxa significantly reduced N_2_O emissions (Figure 5C and Supplementary Figure 3).

## 4 Discussion

### 4.1 Response of gaseous N emissions to green manure incorporation and N fertilizer reduction

The effect of green manure and N fertilization on soil gaseous N emissions is a critical aspect of nutrient management in agriculture (Lyu et al., 2023; Pu et al., 2023; Kumar and Bordoloi, 2024). Our study confirms the complex interactions between green manure types and N application levels in influencing NH_3_ and N_2_O emissions. Specifically, for two consecutive years, OVG incorporation substantially reduced both NH_3_ and N_2_O emissions, by 40.33–44.86% for the entire cotton growth period (*p* < 0.05), compared to NG. This was potentially due to its relatively higher C:N ratio (41.8:1), which decelerates organic matter decomposition, thus limiting the availability of N for microbial processes such as nitrification and denitrification, which contribute to gaseous N emissions (Li S. et al., 2020). In contrast, VVG, with its lower C:N ratio (15.3:1), promotes faster N mineralization and release, leading to higher NH_3_ emissions, despite its moderate reduction effect on N_2_O emissions (Duan et al., 2019; Sun et al., 2021). The differential effects of green manure types suggested that the C:N ratio is a critical factor regulating soil N transformations and subsequent gaseous emissions (Zhang et al., 2024).

A reduction in N fertilization led to substantial decreases in both NH_3_ and N_2_O emissions. Lower N application rates (N2, N1, and N0) resulted in a significant reduction in NH_3_ emissions by 9.95–78.17%, compared to the highest N application rate (N3) over a two -year period. This reduction was largely due to decreased N availability in the soil, which in turn reduced the substrate available for NH_3_ volatilization (Liu et al., 2020). Similarly, N_2_O emissions decreased with reduced N application, and the N2, N1, and N0 treatments significantly lowered N_2_O emissions by 12.27–69.55%, compared with N3, for a two -year period. This trend was consistent with the understanding that reduced soil N levels limit the availability of substrates for nitrification and denitrification processes, thereby reducing N_2_O production (Wang F. et al., 2024).

The significant interaction between green manure incorporation and N fertilizer levels (*p* < 0.05) underscores the importance of these management practices in tandem for regulating gaseous N emissions. The observed synergistic effect suggested that combining N fertilizer reduction with high C-N ratio green manures, such as OVG, is an effective strategy for minimizing greenhouse gas emissions from agricultural soils. This integrated approach not only mitigates the environmental impact of N fertilizer input but also enhanced soil fertility, aligning with the objectives of sustainable agriculture (Wang P. et al., 2024).

### 4.2 Roles of bacterial communities to green manure incorporation and nitrogen fertilizer reduction

The incorporation of green manure and N fertilizer reduction significantly influenced the composition, diversity, community assembly, and co-occurrence networks of soil bacteria. Specifically, green manure incorporation, particularly OVG, significantly enriched Proteobacteria, whereas VVG reduced Acidobacteriota. The observed increase in Proteobacteria under OVG may suggest enhanced nutrient turnover, as members of this phylum are frequently associated with copiotrophic conditions and rapid decomposition of organic materials (Liu et al., 2023). By contrast, the reduction in Acidobacteriota in the VVG treatment aligned with their preference for oligotrophic environments and highlighted the selective impact of green manure on bacterial community composition (Kielak et al., 2016; Liu et al., 2023). Changes in the dominant genera, such as the enrichment of *Skermanella* and *Sphingosinicella* under VVG and OVG, further suggested that specific green manure types can support niche differentiation, potentially benefiting soil function and plant health. N fertilizer reduction also significantly impacted bacterial community composition and diversity. Decreased N levels led to a reduction in Acidobacteriota and an increase in Chloroflexi. Taxa of the phylum Chloroflexi, particularly *Thermomarinilinea,* are known for their metabolic versatility and ability to thrive under nutrient-limited conditions, which may explain their proliferation under reduced N availability (Wu et al., 2024). The decreased abundance of *Usitatibacter* of the phylum Proteobacteria further suggested a decline in taxa that prefer N-rich environments (Wang F. et al., 2024). Although alpha diversity indices did not significantly change across N levels, the compositional shifts indicated that microbial communities adaptively reorganized without significant loss of diversity, which may be critical for sustaining ecosystem functions under reduced N input (Osburn et al., 2023).

Green manure treatments, particularly OVG and VVG, enhanced heterogeneous selection, likely because of the varied C inputs and organic compounds from these green manures, which create diverse microhabitats and resource niches. This resource heterogeneity facilitates niche partitioning, allowing for selective growth of specific bacterial taxa adapted to distinct environmental conditions (Liang et al., 2023). Conversely, N reduction increased homogeneous selection, narrowing the niche breadths and reducing resource diversity. This shift suggested that limited N availability constrains ecological differentiation, imposing uniform selective pressures that streamline community composition. These findings aligned with recent studies that highlight the critical role of nutrient availability and input quality in modulating community assembly through resource competition and niche differentiation (Liao et al., 2024). The selective pressures from green manure incorporation foster a functionally resilient community by promoting taxonomic and functional diversity, while N reduction streamlines community assembly, potentially favoring taxa with efficient nutrient utilization under limited resources (Wang F. et al., 2024). Such interactions between nutrient management practices and microbial assembly processes underscore the need for balanced inputs to support both diversity and functionality in agricultural soils.

Co-occurrence network analysis provided insights into the complexity and resilience of bacterial communities under different treatments. The OVG and VVG treatments produced more complex networks, with higher numbers of nodes and edges, indicating enhanced microbial interactions and potential functional redundancy. This complexity in network structure under green manure treatments reflected the enriched resource diversity and habitat heterogeneity, which may facilitate functional complementarity and cross-feeding among microbes (Fan et al., 2021). By contrast, N reduction led to simpler networks with reduced microbial interactions, reflecting diminished ecological niches and interaction opportunities as N availability decreased (Wang F. et al., 2024). The identification of keystone taxa, such as *Sphingomonas*, *Azohydromonas*, and *Phototrophicus* in green manure treatments, underscored the crucial role of specific bacterial genera in sustaining microbial community structure and ecological function. The higher prevalence of these keystone genera under green manure treatments suggested that the diverse C inputs from organic amendments create more complex ecological niches, allowing for a functionally diverse and resilient microbial network (Zheng et al., 2022). This increased diversity likely enhanced the community’s ability to perform critical ecosystem services, thereby promoting efficient soil nutrient cycling (Xu et al., 2023). Moreover, as N levels decreased, the number of keystone taxa also declined, particularly at the lowest N levels (N0 and N1). Keystone species are integral to maintaining microbial community stability and ecosystem processes (Banerjee et al., 2018). At moderate N levels, keystone genera such as *Longilinea and Litorilinea* (Chloroflexi) were more prevalent. These genera support nutrient transformation processes that are critical under reduced N input conditions. Therefore, moderate N levels maintained a robust microbial network, and the addition of green manure provided functional resilience via the diversification of keystone taxa.

### 4.3 Ecological linkages between soil gaseous N emissions, bacterial community, and soil properties

The interactions between soil properties, bacterial communities, and N emissions, such as NH_3_ and N_2_O, are critical for understanding the ecological dynamics in agroecosystems (Zhang L. et al., 2022). The findings indicate that soil properties play a dominant role in shaping bacterial communities, which in turn influences NH_3_ and N_2_O emissions. Specifically, NO_3_^−^-N and NH_4_^+^-N exhibited strong positive correlations with N emissions, consistent with their function as substrates for nitrification and denitrification processes that generate NH_3_ and N_2_O (Pinheiro et al., 2019). In contrast, MBC exhibited negative loading, suggesting that increased C availability may promote microbial N immobilization, thereby limiting N availability for gaseous emission (Chen M. et al., 2020). This indirect influence of soil properties on NH_3_ and N_2_O emissions through bacterial community modulation underscored the importance of microbes as mediators of soil N transformations. Such interactions aligned with the conclusions of previous studies which indicate that shifts in microbial diversity and structure can substantially impact soil gas emissions by altering microbial metabolic pathways (Guo et al., 2023).

Interestingly, while bacterial communities showed a non-significant direct effect on NH_3_ emissions, they exerted a significant negative effect on N_2_O emissions. The bacterial keystone taxa played a significant role in reducing N_2_O emissions, suggesting that specific microbial species may stabilize community functions under fluctuating N conditions, potentially by enhancing N immobilization or promoting alternative N pathways that reduce N_2_O production. Recent studies have also highlighted the role of keystone microbial groups in maintaining ecological stability and mitigating greenhouse gas emissions under varying environmental conditions (Deng et al., 2024; Liu et al., 2024). Additionally, beta diversity showed a positive loading, indicating that diverse bacterial communities may contribute to N cycle stabilization through functional redundancy, although this effect was secondary to that of keystone taxa (Zhou et al., 2022).

### 4.4 Study limitations and implications

Our results provided valuable insights into the potential of incorporating green manure and reducing N fertilization to mitigate NH_3_ and N_2_O emissions, supporting strategies for sustainable nutrient management in agricultural systems; however, several limitations should be acknowledged. The co-occurrence networks and identification of keystone taxa, along with the presumed interactions among biotic and abiotic factors, were derived primarily from correlational analyses. While the co-occurrence network analysis provided valuable insights into potential microbial associations with N transformation processes, the inherent limitations of correlational approaches precluded definitive causal inferences regarding the direct microbial regulation of NH_3_ and N_2_O emissions. Disentangling direct and indirect microbial interactions in co-occurrence networks impacting N emissions remains challenging (Hirano and Takemoto, 2019; Kishore et al., 2023). Although our results were based on a single-year measurement, the gas monitoring experiment spanned more than 3 years, with consistent trends in N emissions observed annually, aligning with previous findings (Zhang Z. et al., 2022). Future research should validate the driving mechanisms of key bacteria (e.g., *Sphingosinicella* and *Azohydromonas*) in N emissions through bacterial isolation and culture experiments, thereby elucidating microbial regulatory functions in the N cycle. Despite these constraints, our study has important implications for sustainable agriculture. A balanced approach to N fertilization, combined with organic amendments such as green manure can optimize soil microbial networks, thus mitigating NH_3_ or N_2_O emissions and promoting stability and functionality in N cycling.

## 5 Conclusion

Our study confirmed that incorporating green manure with reduced N fertilization effectively regulated gaseous N emissions in cotton fields. Compared with conventional N practices without green manure, OVG coupled with 25% reduction in N fertilization substantially mitigated gaseous N emissions, both for NH_3_ and N_2_O emissions. This reduction was associated with improvements in soil MBC and roles of a group of recruited keystone bacterial taxa in bacterial co-occurrence networks such as *Sphingosinicella*, *Azohydromonas*, and *Phototrophicus*. These findings underscored the potential of green manure and N reduction as synergistic strategies for minimizing agricultural N emissions, These findings revealed the synergistic mechanisms between green manure incorporation and optimized N management for agricultural emission reduction, providing a novel strategy for sustainable N management in agroecosystems. Further research across varying soil types and climatic conditions will be essential to validate these findings and support their broader applicability across diverse agroecosystems.

## Data Availability

The datasets presented in this study can be found in online repositories. The names of the repository/repositories and accession number(s) can be found in the article/Supplementary material.
