# Supplementary material for: Mitigating gaseous nitrogen emissions in cotton fields through green manure and reduced nitrogen fertilization
Source: Front Microbiol. 2025 Jun 2;16:1615142. doi: 10.3389/fmicb.2025.1615142 (PMC12171444; doi:10.3389/fmicb.2025.1615142)
Supplement: Supplementary file 1 [file Data_Sheet_1.docx]

Supplementary Material of

**Mitigating gaseous nitrogen emissions in cotton fields through green manure and reduced nitrogen fertilization**

**
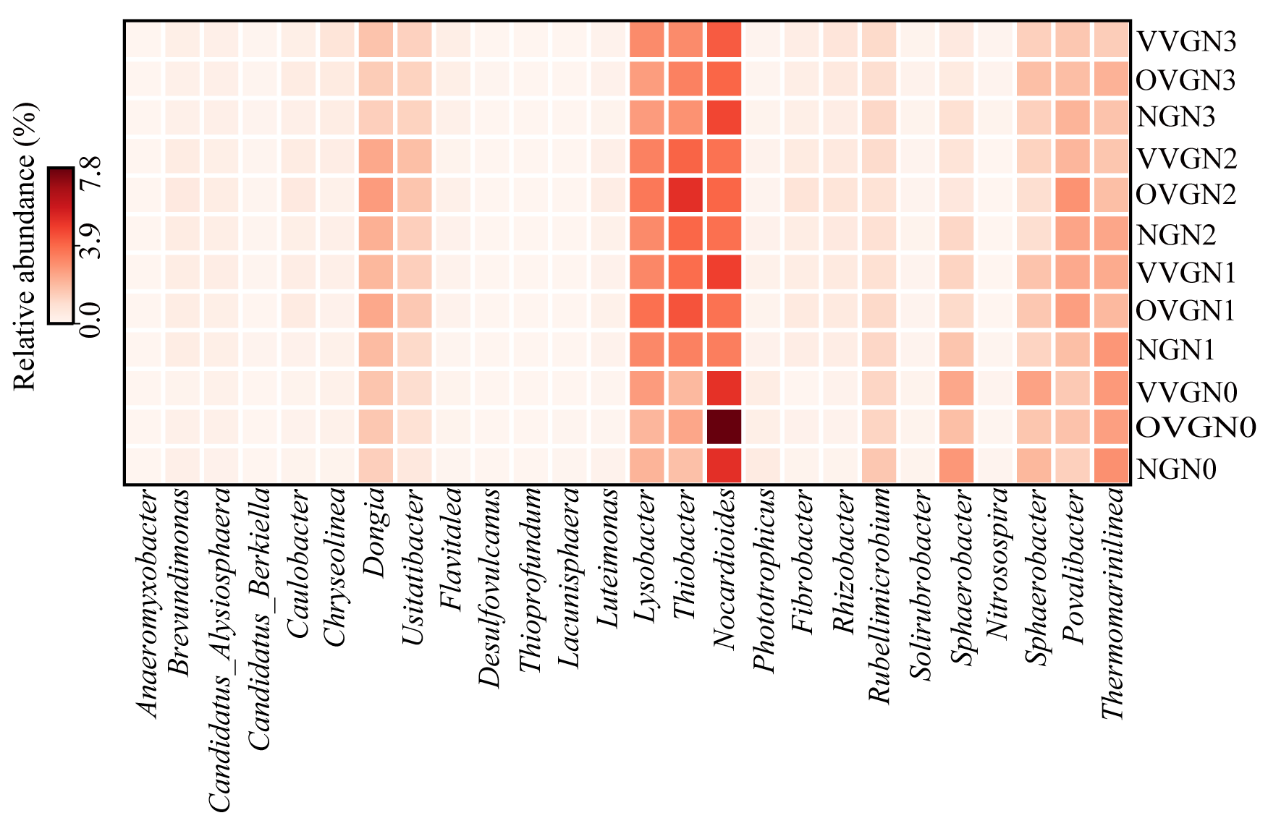
**

**Supplementary Figure 1** Differential bacterial genera responding to green manure incorporation and nitrogen fertilizer reduction. Significant genera identified across different treatments using the Tukey-Kramer test (*P* < 0.05, n = 3).


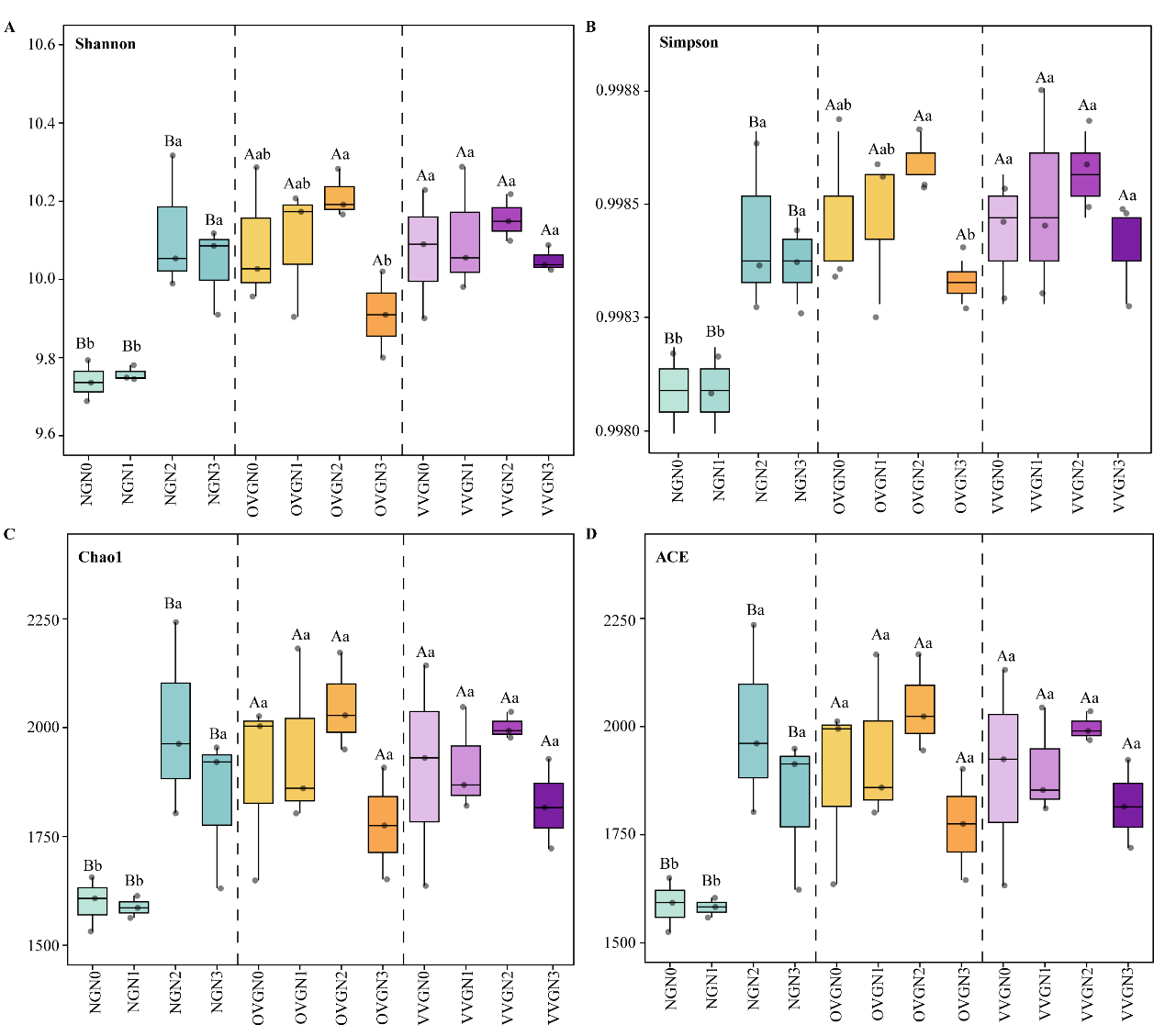
**Supplementary Figure 2** Alpha diversity of bacterial community assessed by (A) Shannon, (B) Simpson, (C) Chao1, and (D) ACE indices. Different letters of the same index indicate statistically significant differences (*P* < 0.05, n = 3) based on Tukey's HSD post-hoc test.


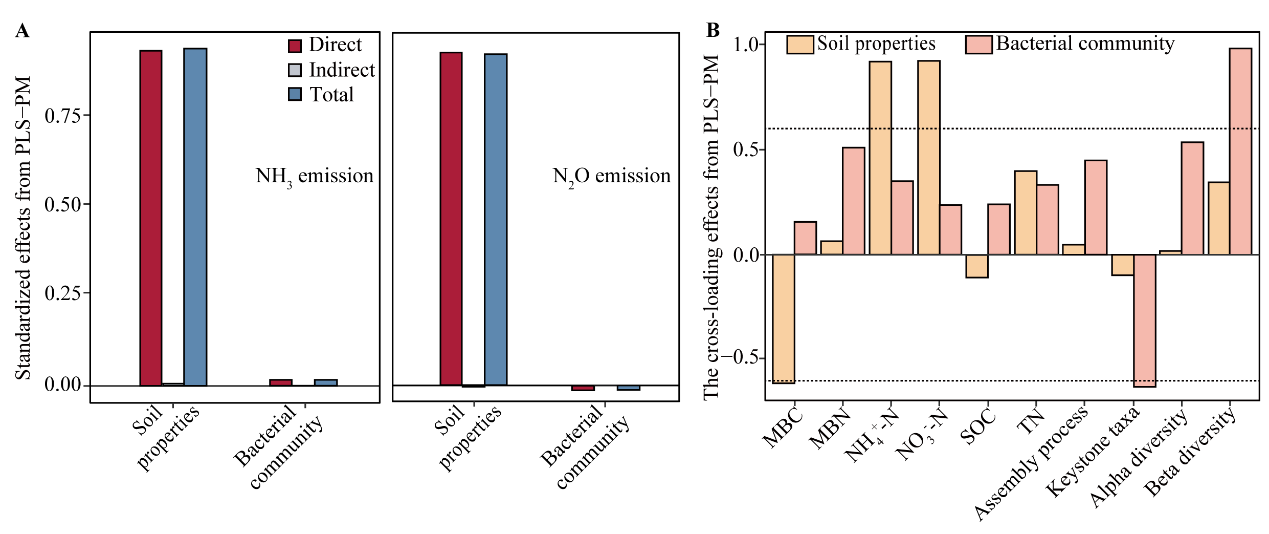


**Supplementary Figure 3** The standardized direct, indirect, and total effects of soil properties and bacterial communities on NH_3_ and N_2_O emissions (A) and cross-loading values from PLS-PM for soil properties and bacterial community metrics (B). Bars represent the loading of various bacterial community attributes and soil properties on latent variables, with factor loadings exceeding 0.6 indicated by the dotted line.

**Supplementary Table 1** Cumulative NH_3_ and N_2_O emission flux under green manure incorporation and nitrogen fertilizer reduction in cotton fields

| **Treatment** | **NH_3_**  **Basal fertilizer cumulative emissions (kg ha^-1^）** | | **NH_3_**  **Topdressing emissions (kg ha^-1^）** | | **NH_3_**  **Total cumulative emissions (kg ha^-1^）** | | **N_2_O**  **Total cumulative emissions (kg ha^-1^）** | | **Total gaseous N emissions (kg ha^-1^）** | |
| --- | --- | --- | --- | --- | --- | --- | --- | --- | --- | --- |
|  | **2022** | **2023** | **2022** | **2023** | **2022** | **2023** | **2022** | **2023** | **2022** | **2023** |
| **NGN0** | 0.50±0.14Ab | 1.74±0.87Ab | 0.58±0.04Ab | 0.07±0.03Ab | 1.08±0.17Ac | 1.81±0.90Ab | 1.25±0.05Ac | 1.06±0.17Ac | 2.33±0.12Ac | 2.87±0.96Ac |
| **NGN1** | 3.27±0.10Aa | 2.43±0.17Ab | 0.94±0.13Ab | 0.60±0.41Ab | 4.21±0.03Ab | 3.03±0.48Aab | 1.74±0.14Ab | 2.34±0.36Ab | 5.95±0.17Ab | 5.37±0.35Ab |
| **NGN2** | 3.11±0.09Aa | 2.73±0.52Ab | 1.64±0.70Aa | 1.49±0.65Aa | 4.75±0.78Aab | 4.22±1.17Aa | 2.11±0.05Aa | 2.62±0.30Aab | 6.87±0.83Aab | 6.84±1.43Aab |
| **NGN3** | 3.50±0.68Aa | 3.96±0.30Aa | 1.74±0.09Aa | 0.33±0.19Ab | 5.25±0.69Aa | 4.29±0.44Aa | 2.38±0.31Aa | 3.12±0.27Aa | 7.62±0.80Aa | 7.41±0.39Aa |
| **OVGN0** | 0.33±0.08Bb | 1.18±0.19Bb | 0.23±0.09ABb | 0.04±0.02Aa | 0.56±0.16Bc | 1.23±0.18Bb | 0.68±0.15Bb | 0.54±0.17Bc | 1.24±0.14Bc | 1.76±0.34Bc |
| **OVGN1** | 1.39±0.20Ba | 2.18±0.51Bab | 0.60±0.12ABb | 0.30±0.09Aab | 1.99±0.10Bb | 2.48±0.60Ba | 0.92±0.17Bb | 0.61±0.11Bc | 2.91±0.12Bb | 3.09±0.49Bb |
| **OVGN2** | 1.68±0.22Ba | 2.10±0.76Bab | 0.75±0.18ABb | 0.70±0.30Aa | 2.43±0.07Bab | 2.79±0.49Ba | 1.43±0.24Ba | 0.91±0.07Bb | 3.86±0.30Ba | 3.70±0.42Bb |
| **OVGN3** | 1.34±0.16Ba | 2.29±0.48Ba | 1.61±0.66ABa | 0.69±0.29Aa | 2.96±0.57Ba | 2.98±0.72Ba | 1.59±0.07Ba | 1.88±0.08Ba | 4.54±0.64Ba | 4.87±0.70Ba |
| **VVGN0** | 1.02±0.48Ac | 1.79±0.48Ab | 0.37±0.07Bb | 0.12±0.01Ab | 1.38±0.52Ac | 1.91±0.49ABb | 1.41±0.15Ad | 0.99±0.01Ab | 2.80±0.52Ac | 2.90±0.49Ac |
| **VVGN1** | 3.31±1.01Ab | 2.46±0.58Ab | 0.81±0.28Ba | 0.42±0.25Aab | 4.12±1.17Ab | 2.88±0.34ABb | 1.83±0.15Ac | 2.76±0.47Aa | 5.95±1.29Ab | 5.63±0.64Ab |
| **VVGN2** | 4.24±0.54Aab | 2.83±0.57Aab | 1.05±0.30Ba | 0.40±0.21Aab | 5.29±0.40Aab | 3.22±0.78ABab | 2.50±0.35Ab | 2.90±0.21Aa | 7.80±0.53Aa | 6.13±0.82Ab |
| **VVGN3** | 4.87±0.85Aa | 3.84±0.91Aa | 0.78±0.13Ba | 0.50±0.19Aa | 5.65±0.79Aa | 4.34±1.00ABa | 2.93±0.11Aa | 3.49±0.57Aa | 8.58±0.68Aa | 7.83±1.23Aa |
| **GM** | ******* | ****** | ****** | **ns** | ******* | ****** | ******* | ******* | ******* | ******* |
| **NF** | ******* | ******* | ******* | ******* | ******* | ******* | ******* | ******* | ******* | ******* |
| **GM*NF** | ******* | **ns** | ***** | ****** | ***** | **ns** | **ns** | ******* | ***** | ***** |

Data are presented as mean ± standard error (n = 3). Uppercase letters indicate significant differences at *P* < 0.05 according to LSD test among different green manure incorporation patterns (GM), and lowercase letters indicate significant differences among distinct N fertilization treatments (NF). NG, no incorporation (control); OVG, incorporation of *Orychophragmus violaceus*; VVG, incorporation of *Vicia villosa*; N3, maximum economic nitrogen rate; N2, 25% nitrogen reduction compared to N3; N1, 50% nitrogen reduction compared to N3; N0, no nitrogen application. Asterisks denote statistical significance at * *P* < 0.05, ** *P* < 0.01, and *** *P* < 0.001; “ns” represents not significant.

**Supplementary Table 2** Keystone taxa of co-occurrence networks under different green manure incorporation and nitrogen fertilizer reduction systems

| **Treatment** | **ASV** | **Relative abundance (%)** | **Network role** | **Phylum** | ***Genus*** |
| --- | --- | --- | --- | --- | --- |
| **NG** | ASV75 | 0.19 | Module hubs | Chloroflexi | *Longilinea* |
|  | ASV225 | 0.07 | Connectors | Myxococcota | *Phaselicystis* |
|  | ASV251 | 0.03 | Connectors | Proteobacteria | *Coralloluteibacterium* |
|  | ASV692 | 0.06 | Connectors | Acidobacteriota | *Vicinamibacter* |
| **OVG** | ASV247 | 0.04 | Module hubs | Chloroflexi | *Kouleothrix* |
|  | ASV514 | 0.03 | Module hubs | Proteobacteria | *Sphingomonas* |
|  | ASV555 | 0.03 | Module hubs | Acidobacteriota | *Holophaga* |
|  | ASV70 | 0.08 | Connectors | Chloroflexi | *Phototrophicus* |
|  | ASV397 | 0.03 | Connectors | Proteobacteria | *Azohydromonas* |
|  | ASV781 | 0.03 | Connectors | Gemmatimonadota | *Roseisolibacter* |
| **VVG** | ASV70 | 0.09 | Module hubs | Chloroflexi | *Phototrophicus* |
|  | ASV688 | 0.03 | Module hubs | Gemmatimonadota | *Gemmatimonas* |
|  | ASV114 | 0.06 | Connectors | Bacteroidota | *Flavobacterium* |
|  | ASV177 | 0.02 | Connectors | Thermomicrobiota | *Sphaerobacter* |
|  | ASV378 | 0.04 | Connectors | Chloroflexi | *unclassified_Anaerolineae* |
|  | ASV528 | 0.05 | Connectors | Proteobacteria | *Arboricoccus* |
|  | ASV555 | 0.02 | Connectors | Acidobacteriota | *Holophaga* |
|  | ASV645 | 0.03 | Connectors | Acidobacteriota | *Vicinamibacter* |
|  | ASV669 | 0.02 | Connectors | Actinobacteriota | *Cellulosimicrobium* |
|  | ASV801 | 0.03 | Connectors | Thermodesulfobacteriota | *Thermodesulforhabdus* |
| **N0** | ASV79 | 0.06 | Connectors | Proteobacteria | *Devosia* |
|  | ASV688 | 0.03 | Connectors | Gemmatimonadota | *Gemmatimonas* |
| **N1** | ASV243 | 0.08 | Module hubs | Proteobacteria | *Azohydromonas* |
|  | ASV660 | 0.03 | Module hubs | Bacteroidota | *Parasegetibacter* |
|  | ASV731 | 0.08 | Module hubs | Chloroflexi | *Longilinea* |
|  | ASV312 | 0.04 | Connectors | Bacteroidota | *Rhodocytophaga* |
|  | ASV770 | 0.03 | Connectors | Proteobacteria | *Frateuria* |
|  | ASV925 | 0.03 | Connectors | Acidobacteriota | *Vicinamibacter* |
| **N2** | ASV142 | 0.09 | Module hubs | Proteobacteria | *Wenzhouxiangella* |
|  | ASV428 | 0.10 | Module hubs | Bacteroidota | *Chitinophaga* |
|  | ASV555 | 0.02 | Module hubs | Acidobacteriota | *Holophaga* |
|  | ASV208 | 0.04 | Connectors | Proteobacteria | *Paraburkholderia* |
|  | ASV336 | 0.08 | Connectors | Thermodesulfobacteriota | *Desulfuromonas* |
|  | ASV667 | 0.03 | Connectors | Chloroflexi | *Litorilinea* |
| **N3** | ASV312 | 0.04 | Module hubs | Bacteroidota | *Rhodocytophaga* |
|  | ASV325 | 0.05 | Module hubs | Acidobacteriota | *Luteitalea* |
|  | ASV378 | 0.03 | Module hubs | Chloroflexi | *Dehalogenimonas* |
|  | ASV99 | 0.08 | Connectors | Proteobacteria | *Caldimonas* |
|  | ASV341 | 0.05 | Connectors | Acidobacteriota | *Luteitalea* |
|  | ASV349 | 0.04 | Connectors | Bacteroidota | *Ohtaekwangia* |
|  | ASV360 | 0.08 | Connectors | Thermodesulfobacteriota | *Desulfonatronum* |
|  | ASV442 | 0.06 | Connectors | Proteobacteria | *Elstera* |
|  | ASV447 | 0.07 | Connectors | Actinobacteriota | *Iamia* |
|  | ASV692 | 0.09 | Connectors | Acidobacteriota | *Vicinamibacter* |
|  | ASV833 | 0.03 | Connectors | Chloroflexi | *Ornatilinea* |

NG, no incorporation (control); OVG, incorporation of *Orychophragmus violaceus*; VVG, incorporation of *Vicia villosa*; N3, maximum economic nitrogen rate; N2, 25% nitrogen reduction compared to N3; N1, 50% nitrogen reduction compared to N3; N0, no nitrogen application.
